# Retooling Computational Techniques for EEG-Based Neurocognitive Modeling of Children's Data, Validity and Prospects for Learning and Education

**DOI:** 10.3389/fncom.2019.00004

**Published:** 2019-02-18

**Authors:** Amedeo D'Angiulli, Peter Devenyi

**Affiliations:** ^1^Neuroscience of Imagination Cognition and Emotion Research Lab, Ottawa, ON, Canada; ^2^Department of Neuroscience, Carleton University, Ottawa, ON, Canada; ^3^Child Studies Programme, Institute of Interdisciplinary Studies, Carleton University, Ottawa, ON, Canada

**Keywords:** EEG, Event-Related Potentials, neurocognitive modeling, visual attention, word comprehension, personalization, inclusive education, educational neurofeedback

## Abstract

This paper describes continuing research on the building of neurocognitive models of the internal mental and brain processes of children using a novel adapted combination of existing computational approaches and tools, and using electro-encephalographic (EEG) data to validate the models. The guiding working model which was pragmatically selected for investigation was the established and widely used Adaptive Control of Thought-Rational (ACT-R) modeling architecture from cognitive science. The anatomo-functional circuitry covered by ACT-R is validated by MRI-based neuroscience research. The present experimental data was obtained from a cognitive neuropsychology study involving preschool children (aged 4–6), which measured their visual selective attention and word comprehension behaviors. The collection and analysis of Event-Related Potentials (ERPs) from the EEG data allowed for the identification of sources of electrical activity known as dipoles within the cortex, using a combination of computational tools (Independent Component Analysis, FASTICA; EEG-Lab DIPFIT). The results were then used to build neurocognitive models based on Python ACT-R such that the patterns and the timings of the measured EEG could be reproduced as simplified symbolic representations of spikes, built through simplified electric-field simulations. The models simulated ultimately accounted for more than three-quarters of variations spatially and temporally in all electrical potential measurements (fit of model to dipole data expressed as *R*^2^ ranged between 0.75 and 0.98; *P* < 0.0001). Implications for practical uses of the present work are discussed for learning and educational applications in non-clinical and special needs children's populations, and for the possible use of non-experts (teachers and parents).

## Introduction

The primary goal of this paper is to report on continuing research on the building of cognitive models of the internal mental or brain processes of children by using the measurements of electroencephalographic (EEG) data in order to validate the models constructed. Furthermore, as a secondary but consequentially related goal, this paper explores prospective, possible implications in terms of designing useful neuro-technologies for learning and education, with specific consideration of two major themes currently debated in education: personalization and inclusion. The presented data were collected from neuropsychological experiments conducted with children from 4 to 6 years of age, which measured their visual selective attention and word comprehension in two separate computerized tasks. The collection and analysis of Event-Related Potentials (ERPs) from the data of scalp EEGs allowed for the identification within the cortex of dipoles as the sources of electrical activity.

In over fifty years of research, psychology, neuroscience, cognitive science, and other allied disciplines have clearly shown that to specify the neural/mental processes involved in a task from a human agent, behaviorally manifested differences in the extent of responses and their latency are necessary but not sufficient (Frank and Badre, 2015). Further steps are required to specify which structures and which functional pathways are putatively involved (Griffiths, 2015). In principle, analysis of verbal protocols (Ericsson and Simon, 1993), and other forms of verbal reports (see Runge et al., 2017) could be used to build converging validity for neurocognitive models using the “phenomenology-neural-behavior triangulation” (see Flanagan and Dryden, 1998). However, determining all these elements in young (i.e., infants and preschoolers) children escalates complexity further. This is where the present study, involving neuro-computational modeling (henceforth shortened as neurocognitive modeling), comes into play. A background question permeating this work concerns how much reduction is tolerable in order to achieve models that could one day be relatively easily implemented for real-world, practical applications for learning (ideally by users such as, for example, educators, teachers, and parents or the learners themselves, the children). For these reasons, the present work assumes the very pragmatic tactic of combining already existing and validated computational tools in a novel way.

**Figure 1:**
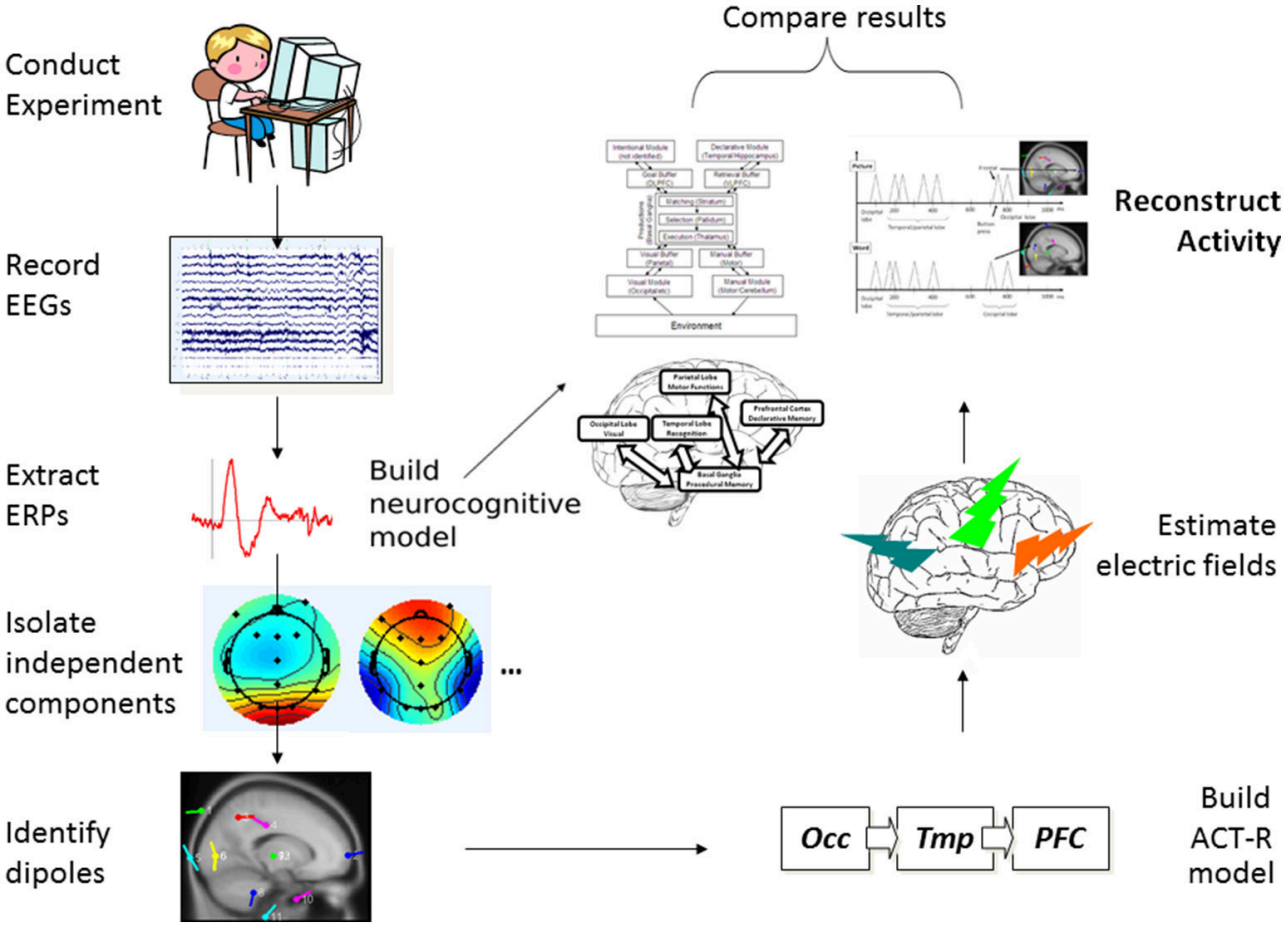
Process flow representation of the present study.

As the starting point, the guiding working model which was pragmatically selected for investigation was the established and widely used ACT-R cognitive modeling architecture (ACT-R research Group, 2019). The MRI-based circuitry covered by ACT-R (see **Figure 3**) overlaps considerably with the circuitry considered and studied by many neuroscience research programs (see Borghi et al., 2013) independently from computational and modeling applications. The advantage of such an approach is that the correlates considered could in turn be modeled and verified as functional pathways through the building blocks of ACT-R. And of course, the results of the modeling can feed back to inform theory about neurocognitive functions and structures (for examples see Polk and Seifert, 2002). This cycle informed the design of the present study and is represented graphically in Figure 1.

As represented in the process flow of Figure 1, from the initial collection of children's EEG data in an experiment involving two tasks measuring aspects of different but related cognitive processes, using a type of Independent Component Analysis (Jung et al., 2001) we extracted and isolated single-trial ERPs and identified dipoles, indicating their likely sources within the cortex and other (Subcortical) parts of the brain. We then mapped this information onto a generalized ACT-R neurocognitive model with multiple interactive components. At the same time, from scalp single-trial ERPs from the three key brain cortical areas postulated in ACT-R, we simulated simple spike representation using a reductionist electric-field estimation procedure, which allowed us to reconstruct the cortical activity over time for the two tasks as it would happen in each individual trial. Finally, we compared the neurocognitive model and the reconstructed activity to assess whether the two types of results could be coherently integrated as a whole product.

The need for relatively precise spatial localization and connectivity in the model was further insured by adopting basic neural-spiking simulation techniques to be able to confirm the following: (1) the time latency of ERP activity linked to the identified patterns of activation within the ACT-R architecture; and (2) validity of the postulated meaning of the ERP components (i.e., higher amplitude reflecting the relevant increased neural recruitment in the involved structures). Our expectation was that the combination of already popular and widely used modeling approaches would provide converging evidence supporting the hypothesized processes of attention and acquisition of the word meanings and implicate a network of connections overlapping in key cortical networks, in particular, those involved in the occipital-temporal-prefrontal long-range connections (see Table 1, and Figure 1) shared by the two types of tasks in developing brains (see D'Angiulli et al., 2015). For the latter reason, in this paper we focused our analyses on the selected electrodes of interest corresponding to the main cortical areas involved in those neural networks.

**Table 1:** Times and locations of modules in ACT-R model for selective attention and PPVT tasks.

	**Region(s)**	
**Time (ms)**	**Viewing pictures (attention and PPVT)**	**Word verification**
100	Occipital/Basal, Bilateral	Bilateral Occipital/Right Frontal
125	Parietal/Basal, Bilateral	Parietal/Basal, Bilateral
170	Right Occipital/Left Frontal	Right Occipital/Left Frontal
220	Basal, Bilateral	—
280	Parietal/Frontal, Bilateral	Left Frontal/Left Occipital/Right Temporal
320	Right Parietal/Right Temporal	Left Frontal/Left Occipital/Right Temporal
380	Left Parietal	Left Frontal/Left Occipital/Right Temporal
690	Left Occipital/Left Basal	Left Occipital
850	—	Right Occipital/Bilateral Temporal

## Materials and Methods

In the following “Experiment” subsection, we describe two behavioral tasks with a sample of young children: one measured the activation of the sensorimotor and perceptual systems engaged in a visual selective attention task, the other task measured linguistic-conceptual and semantic memory systems engaged in a word-verification task. Both tasks were part of a large developmental cognitive neuroscience research program, and were published as primary data analysis reports elsewhere (Van Roon and D'Angiulli, 2014; D'Angiulli et al., 2015). In what follows, we provide a summary of the essential steps to illustrate how the entire protocol can be replicated. However, readers interested in more details on the human experimentation side should consult the cited reports. ERPs were extracted from continuous EEGs time-locked to the task stimuli, to identify the sources of electrical activity within the cortex known as dipoles. Subsequently, in the subsection titled “Neurocognitive Modeling,” we describe how the results of the experimental tasks were used as secondary data analysis and manipulation to build neuro-computational models that could reproduce localizations, dynamic connectivity among areas, patterns of neural spiking, and timings of measured EEG (see Figure 1). An important point of difference with the previously published results is that we present here novel analysis focusing on selected samples of the best instances of observed *single-trial ERPs* (across different subjects) as identified by a type of ICA. That is, in creating the models we did not use grand averages of already-averaged ERPs across all trials.

### Experiment

#### Participants

Participants were initially selected from a prospective cohort of children recruited in the context of a separate, non-overlapping, larger research program on early development screening (D'Angiulli et al., 2009). Based on the extensive developmental literature (Bornstein and Lamb, 2011) and given the scope of our study, we identified as the optimal target developmental period the one corresponding to the age range of 4.5–6.5 years. To recruit the initial pool of participants, an information package was distributed to all parents whose children attended the same daycare of a middle-sized Canadian city. This study was approved by the institutional research ethics boards of Thompson Rivers University and Carleton University in accordance with the 1964 Declaration of Helsinki ethical standards and the Tri-Council Policy Statement (http://www.pre.ethics.gc.ca/pdf/eng/tcps2-2014/TCPS_2_FINAL_Web.pdf). Parents signed a consent form and completed a brief questionnaire on demographic and socioeconomic information about their family, including a clause to consent to this follow-up study including collecting EEG, and behavioral and cognitive measures from their children. Materials explaining what was involved were included in the package and presented at the daycare to teachers and parents during small information sessions. Thus, only general information about the present study was provided to our target families and children. Hypotheses and purposes of the study were only given (verbally to children and through a written take-home page to parents) at debriefing after the study but not at the recruitment stage. After screening for the families' socioeconomic and demographic background information and individuals' daycare records, the prospective participants were matched by age, gender, ethnicity, reported health/physical development and “computeracy” (ownership and use of internet and computers, including video gaming). Thirty families were then re-contacted by mail, of which seventeen returned completed and signed consent for the present study. Children were given a gift card of $5 for their participation and also received a book of stickers at the end of the study. Written parental informed consent and the children's active assent was obtained according to a protocol approved by research ethics boards from all of the involved institutions.

The final sample of 13 children [nine boys; four girls; *mean age* (*SD*) = 5.10 (0.75)] was obtained after exclusion of three (female) participants from the initial sample of 16 (two children had an insufficient number of artifact-free or artifact-corrected usable EEG data and/or did not meet the minimal required performance level (accuracy >75%) in one task, hence their data were discarded after preliminary diagnostic analysis). Following strict inclusion criteria, participants were carefully selected to represent, despite some age variation, a relatively homogeneous group of healthy, typically-developing children. The participants scored all within 0.5 standard deviations from the mean on the following standardized age-normed control measures: parents completed the *Behavioral Rating Inventory of Executive Function—Preschool Version (BRIEF-P)* from Psychological Assessment Resources (PAR), Inc. (Gioia et al., 2005); and the preschool Child Behavioral Checklist (CBCL/1½−5 years; Achenbach, 2009). In addition to the above measures, the participants exceeded expectations in the *Early Development Instrument* (Janus et al., 2007) in all developmental domains (i.e., physical health and well-being; social knowledge and competence; emotional health and maturity; language and cognitive development; communication skills and general knowledge).

Furthermore, according to parent reports and daycare records, the participants were typically-developing children with no history of medication or referral to disability assessment or services. All were Caucasian with normal or corrected-to-normal vision and no hearing or other known sensory impairments. The children lived in the same neighborhood, corresponding to the same catchment area for the daycare center they attended. All children were from middle-upper class family socioeconomic backgrounds.

#### Apparatus and Procedures

##### Behavioral tasks

**Figure 2:**
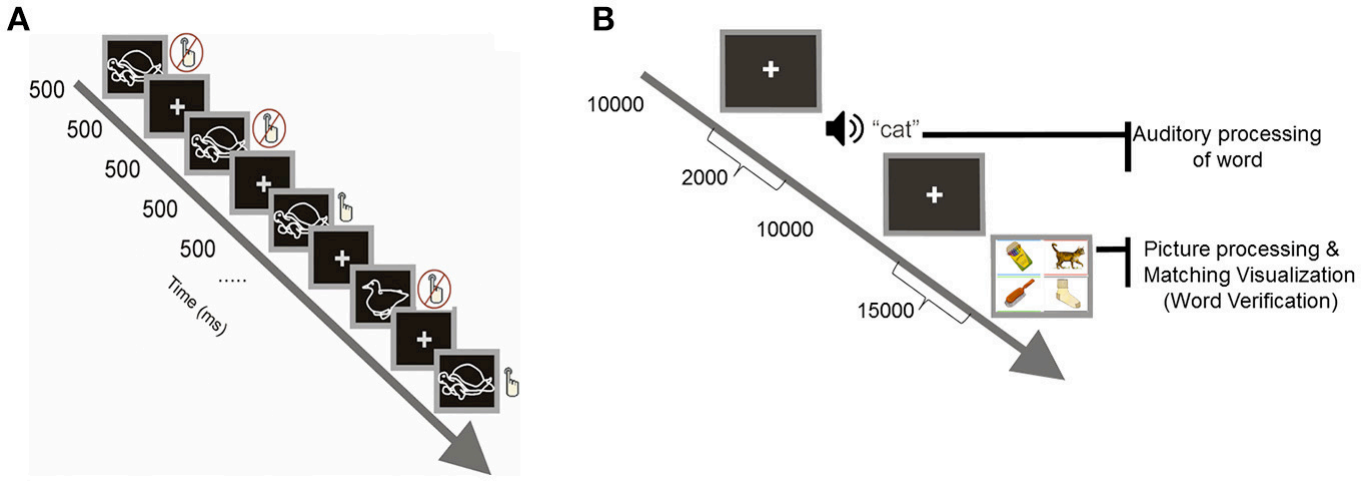
**(A)** Visual selective attention task. **(B)** Computerized version of the Peabody Picture Vocabulary Test.

For the visual selective attention task, the method followed the standard protocol of Akshoomoff (2002) represented in Figure 2A. For this task, children viewed a computer screen which displayed either a picture of a duck or a picture of a turtle that remained on the screen for 500 ms and was followed by a 500 ms ISI. Children were instructed to watch the computer screen and, every time they saw a duck, to push a button, and not to push the button if they saw something else (this requires that children ignore irrelevant stimuli while paying attention to the target stimulus). For one quarter of the trials a duck was displayed, and for the remaining three quarters a turtle was displayed. Each child was given 12 trials or practice periods, followed by 150 trials each.

The task of *word verification* was a computerized version of the standard Picture Peabody Vocabulary Test (PPVT-III), which measures receptive vocabulary and word comprehension (Dunn and Dunn, 2007). The test includes 19 sets, each set includes 24 items: 24 target words presented aurally and 24 corresponding displays containing four-color pictures arranged on the screen, with each picture having a rectangular frame with a different color (spatial layout and colors of frame were randomly shuffled from trial to trial). For each trial and item in a stimulus set, children were seated in front of a computer and heard a word over insert headphones (with sound set at 70 dB). They were then asked to decide which one of the four pictures on the computer screen corresponded to the target word (see Figure 2B). Children were instructed to verify the meaning of the target spoken word by selecting the picture that best illustrated its meaning by clicking on a response keypad having four buttons with colors matching the color-coded frames inscribing the stimulus pictures. All children were instructed to consider all the buttons appropriately to give the correct manual response. The words were prerecorded from the voice of an English-speaking female experimenter at a rate of 250 Hz. Each new trial was self-initiated, upon pressing any button in the response keypad. A set was completed successfully unless three consecutive words were incorrectly verified; in this event, the test was discontinued and the particular set was considered the maximum performance level assigned to the subject.

The stimulus sets are arranged in order of increasing abstractness and complexity as operationally reflected in difficulty so that the task can be calibrated to the participant's appropriate vocabulary level (norm-based critical range). The strong relationship of the type of processes measured by the PPVT and language comprehension has been well-documented (e.g., Carroll, 1993; Kamil and Hiebert, 2005). Accordingly, correlations between PPVT and kindergarten language comprehension are typically very strong (*median r* > 0.65, see Dunn and Dunn, 2007). Thus, performance on PPVT is very unlikely to reflect just shallow linguistic processing in preschool children. This is also abundantly confirmed by overwhelming evidence in the context of aphasiology, intelligence, and clinical neuropsychology literature in both children and adults—research fields in which, at least for the last three decades, the PPVT has been used as a criterion measure of semantic elaboration.

In both tasks, the children were all tested individually in a sound-proof electromagnetically-shielded EEG booth. Each child was positioned in front of the computer so that his or her eyes were ~38 cm from the screen. Children were reminded of instructions or could communicate with experimenters and attending parents in the adjacent control room through an intercom speaker system (parents and experimenters had a back view of the child through a window but also had a frontal and facial view through a Bluetooth camera). The children were reminded of the importance of not speaking, moving/turning their head, clinching teeth, or blinking soon after they had given their manual response and before initiating a new trial. Each task required five minutes for completion.

#### EEG Data Acquisition and Recording Procedures

The EEG was recorded with EEG Quick caps with Ag-AgCl electrodes (Compumedics Neuroscan, Charlotte, NC, USA). Each participant had 11 Ag-AgCl electrode sites (Cz, F3, F4, Fz, O1, O2, Oz, P7, P8, Pz, T7, and T8) applied according to the 10–20 system of electrode application (Nuwer et al., 1998) as used previously (e.g., D'Angiulli et al., 2008, 2013). The decision of having the number of electrodes smaller than 32 was dictated by previous work and pilot studies in children of similar ages, showing no critical loss of reliability in source analysis results (Griffiths et al., 2011). All electrodes were referenced to nose tip. Impedances were kept below 5 kOhms. The vertical electrooculograms (VEOG) were recorded from two split bipolar electrodes on the left and right supraorbital ridge (VEOGU, L and R) as well as the left and right zygomatic arch (VEOG, L and R). The signal from the electrodes was amplified and digitized by a SynAmps2 and a SCAN™ 4.3 EEG system (Compumedics Neuroscan, Charlotte, NC, United States), with filter settings at 0.15 Hz (high pass) and 100 Hz (low pass). The data from all channels were digitized online at a sampling rate of 1,000 Hz.

##### EEG artifact reduction

Ocular artifact reduction was conducted through the eye-movement correction included in the EEGlab package (Delorme and Makeig, 2004). To verify, confirm reliability, and validate our procedure, we correlated our edited data to the data obtained with two additional independently conducted procedures: a manual eye-movement rejection based on visual-score scanning procedure, and on the eye movement reduction algorithm devised by Semlitsch et al. (1986), which consists of constructing an average artifact response and then subtracting it from the EEG channels on a sweep-by-sweep, point-by-point basis. The agreement between the edited data with our procedure and the two confirmatory procedures was high (*r* = 0.89 with artifact rejection and *r* = 0.95, both *p* < 0.0001).

##### General ERP processing and analysis

In this section we describe procedures and analysis parameters that did not vary depending on the nature of the EEG/ERP data, the more specific approaches to the data relative to each subset of tasks are described in the relative sections of the results.

The electrode locations were mapped using the EEGLAB BESA standard-10-5-385 cap model. Each participant's EEG was epoched (200 ms pre-stimulus and 1,000 ms post-stimulus) and averaged with respect to the event of interest, which acted as the anchor for the epoching (the stimulus or 0 ms mark). For the attention task, the considered epoch was anchored on the presentation of the duck or turtle. For the PPVT task, there were two types of epochs: one anchored on the presentation of the word and the other anchored on the subsequent presentation of the four-picture display. Baseline correction was based on the 200 ms pre-stimulus interval.

The analysis of the EEG data was conducted using EEGLAB software from the University of California, San Diego, which runs on the proprietary software MATLAB (Delorme and Makeig, 2004). Event-Related Potentials (ERPs) were then derived from the continuous EEG recordings using two complementary averaging techniques: (1) *Grand averaging* of averaged ERPs across subjects; (2) *averages of single-trial* ERPs across subjects. Performance accuracy rates (>75%) insured that the children carried out the tasks at threshold in pressing the button when appropriate.

For each task, the quantification of the effects was based on maps representing normalized averaged scalp electrical activity (see below), as well as on essential analysis including separated focused contrast analyses using *Z* (standard normal deviate) tests or *t*-tests. The latter procedures were used to calculate the mean standardized difference (in micro-volts) needed in each electrode location in order for the neural activation patterns to be significantly different from one another; such differences can be directly interpreted as effect sizes in the same meaningful metric (Shadish and Haddock, 1994). Contrasts between mean amplitudes were conducted just for the time windows of interest but took into account standard deviations and standard errors of the baseline mean across the entire ERP epochs. Additionally, for ease of interpretation, some of the standardized mean differences valid for all the simultaneous multiple comparisons between types of events are indicated in the depiction of the ERP waveforms in the Figures. *p*-values were corrected for multiple comparisons using the Simes-Bonferroni procedure (Simes, 1986).

### Neurocognitive Modeling

In this section, we describe methods used for building the neurocomputational models (neurocognitive model, in short) of visualization correlates for both the attention and the word-verification tasks we used. The observed EEG/ERP results from the two tasks were used to build models to identify sources of task-related ERP activity (electrical energy), and then to reproduce the pattern and timings of measured ERP peaks.

#### Independent Component Analysis

Further analysis consisted of Independent Component Analysis (ICA) of single-trial ERPs (Jung et al., 2001) as well as subsequent ICA for components of ERPs. This technique mathematically determines sets of discrete separate functions that may efficiently explain all measurements as signals which are maximally independent (the FASTICA algorithm was used, Hyvärinen and Oja, 1997). As an example, a single middle occipital area was discerned from the initial simultaneous reaction of three posterior electrodes. While the location and the timing of components can be calculated with ICA, a magnitude which is absolute cannot be estimated similarly, as there inherently exists an ambiguity between component magnitude and attenuation from it to the point of measurement.

#### ACT-R Framework

**Figure 3:**
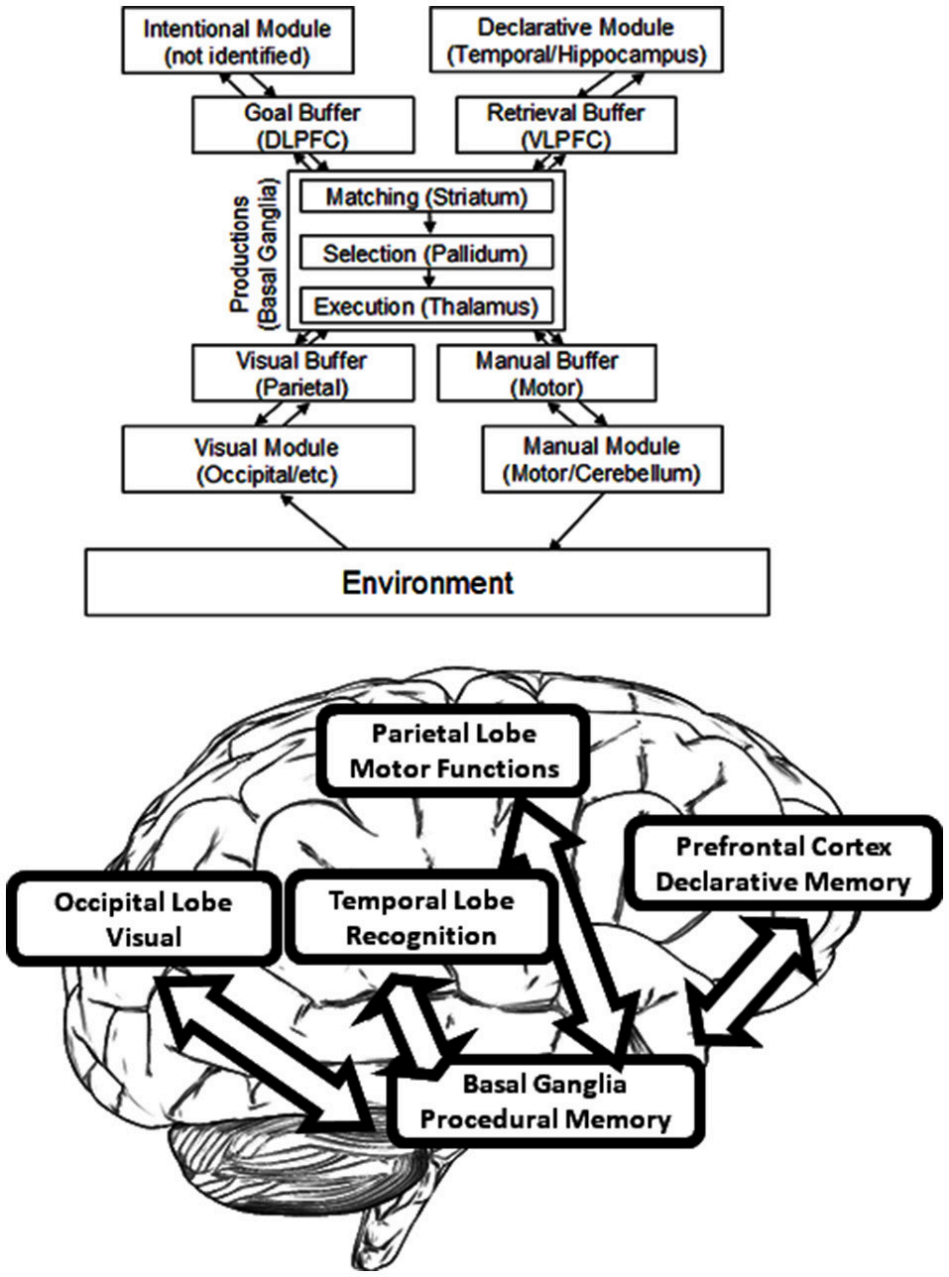
The organization of information in ACT–R.

ACT-R, as developed by John R. Anderson, provides a system for modeling that is commonly used in cognitive science (Anderson and Lebiere, 1998). By this architecture, cognition arises from parallel interactions of modules which are independent. Procedural memory is modeled as a system of production by ACT-R, and specifically one of rules—namely, rules of if/then. A system of buffers and chunks manages communication both to and from the Procedural Model (see Figure 3). In ACT-R, chunks are composed of short lists of information which are predicated (i.e., a chunk could so represent the word “dog” as “Is a”:*dog*, “Name”:*Fido*, “Color”:*brown*, “Size”:*large*). A buffer can contain only one chunk at a time. At least one buffer exists for every module, and therefore a buffer that is visual, and another that is auditory, and another that is declarative, and so forth. Buffer instructions are received by the modules, which send their own results of activity to the buffers. Altogether, buffers may be regarded to form the working memory; they can alternately be considered to represent the current task context. When an “if condition” matches the buffer content, productions are said to “fire.” Buffer content is altered by the part of production known as “then.” Each production requires 50 ms, and productions can fire only one-by-one. Module functions serve to determine the time required of each module to return a result. If for example a specific memory is requested by a production from the Declarative Memory Module, a stronger memory will deliver the results sooner. ACT-R therefore renders strong predictions about internal events.

The standard version of ACT-R was written in LISP. However, we adopted Python ACT-R, which is a recent re-implementation of the architecture (Stewart and West, 2005). This supports most of the functions of ACT-R release six while allowing programming in a more compact and accessible syntax using the Python language.

#### Module Localization

The term “module” is here defined as a function which is local to some area and which also links with a given task process (as distinct from the language modules of Chomsky or the domain-specific modules of Fodor) but which is similar to the generalization offered by Kosslyn (1994). Functional Magnetic Resonance Imaging (fMRI) has accounted for much of the research as it links ACT-R module activity to areas specific in the brain. [The relevant papers can be found on the website for ACT-R (ACT-R research Group, 2019). Those estimates for module location which are proven the best are found listed in (Anderson and Byrne, 2004). In addition, an exhaustive review of these brain area functions can be found in Anderson (2007)]. As an example, the central coordinator for productions is determined to be the caudate of the basal ganglia. Hippocampal control is responsible for declarative memory, whereas attention toward conflicting stimuli is controlled by the anterior cingulate cortex. Declarative memory finds its support from the frontal cortex, whereas visual processing occurs within the occipital lobe along with additional processing in the parietal lobe (Figure 3).

FMRI is ideal for the use of localization. It is, however, generally recognized as being too slow for the detection of events as they would fall within the timeframe of ACT-R. Anderson has avoided this limitation by electing to model the prospective BOLD response in accordance with module activation time course (Anderson et al., 2003), yet this approach nonetheless involves a delay mismatch in real time, as the recording of activation lags behind the processing of cognition by some seconds. And even the so-called event-related fMRI still has a time resolution that is effectively inferior to the one obtained by EEG measurements. Herein, we intended to explore with EEG for much the same reasons, so as to capitalize on the superior time (millisecond) resolution of ERPs. (For a different, but successful example of ERP-based ACT-R modeling, see Cassenti, 2007; Cassenti et al., 2011).

#### Dipole Location

Dipolar analysis was applied for identifying the location of areas of the brain as indicative of the origin of the signals. “Dipole” is a term in physics that refers to one pair of charges which are closely spaced, one being positive and the other being negative. The dipole can create an electrical field, or voltage, at a given distance depending upon the strength and the orientation. One section of the brain can possess many thousands of neurons oriented in a single direction and firing all at once. It may represent a cortical column, a lower structure nucleus, or a ganglion belonging to the basal ganglia, for example. In their firing. these neurons produce voltage, to be measured as EEG in surface scalp electrodes (see Onton and Makeig, 2006). In EEGLAB, the component titled *DIPFIT* was employed for the estimation of a set of dipoles in both single-trial and average data of ERP to explain the independent components which were extracted. The dipole is defined as a region of the cortex wherein many thousands of neurons act in parallel such that their total electrical field amounts to the scalp measurement of EEG. DIPFIT regularly locates one or perhaps two dipoles for each specific region as it appears to have produced the independent individual components in each single trial. The MRI-based spherical head model with standard Tailarach coordinates appropriate to age as of EEGLAB was chosen.

#### Electric-Field Modeling

The next stage was to create a model that reproduced the average ERP activity measured across participants using extraction of single-trial ERPs. An ACT-R model of the process would, at minimum, predict that the visual module (occipital) would be activated by the displayed picture and would place a representation of the picture in the visual buffer (parietal). Next, the “parietal” representation would be used to retrieve the instruction about what to do for that animal from declarative memory (temporal), which in turn would be placed in the declarative memory buffer (frontal). For our purposes, the model was primarily built to reproduce the electrical activity measured rather than behavioral results.

In the neurocognitive model, each module was assumed to generate between one and two dipoles in the dipole-fitting stage of location identification for the simulation of electrical activity. It was believed that the module produced its electrical energy in the rising and falling of a wave. For the purpose of modeling, there was an assumption of a basic triangular wave, with its peak at the module center (simplified spike model). The resultant electrical field or voltage was thereupon calculated at the surface head of each electrode as the total sum contribution of the individual dipoles. Since the peak activities of the components occurred at different times in the observed data, it was not necessary to add the effects of more than one dipole at a given time.

**Figure 4:**
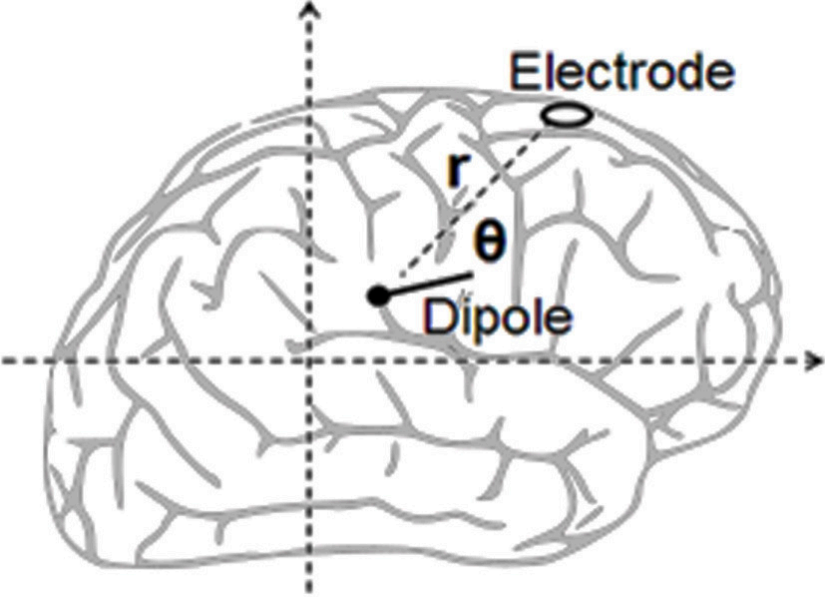
Calculating an electric dipole field.

- The estimation of independent dipole effect reads as below (see Figure 4): The square of the distance (r) from electrode to dipole as obtained from Pythagoras is calculated.
- The square of the distance (r) from electrode to dipole as obtained from Pythagoras is calculated.
- The electrode potential of the dipole is calculated from Coulomb's law (i.e,: k × p × cos(θ)/r^2^), where p equals the dipole strength while k remains constant. It is unnecessary to determine the value of the constant as the models employ relative magnitude.
- Lastly, ERP signal simulations were compared to experimental measurements.

Elsewhere, we have provided proof-of-concept demonstrating that the above method can be used consistently to describe internal neural activity (Griffiths et al., 2011). In this paper, we extend that preliminary work by showing that the set of building blocks are stable across diverse tasks and can be used to reproduce results for further tasks.

## Results

### Behavioral Data

Descriptive analysis on the behavioral data showed that for the attention task, accuracy was very high (*M* = 89.85%; *SD* = 5.03) and relatively rapid (*mean RTs* = 745 ms, *SD* = 366.17). Age correlated significantly with accuracy [*r*_(13)_ = 0.61, *p* < 0.05] but not with RTs. On the longer PPVT task, children took on average 3.5 s (*SD* = 0.78) to respond from when the picture set was presented. Similarly as with the attention task, accuracy was relatively high as all children completed successfully between 2 and 6 sets of target words, with most children being within ±1 SD from the mean in terms of age-normed standard scores. There was no correlation between age and performance accuracy or RTs on the PPVT test.

### ERP Data

Constrained by our modeling approach (see sections Dipole Location and Electric-Field Modeling), our ERP analysis focused selectively on the electrodes corresponding to the brain areas hypothesized and tested by ACT-R. We therefore report only significant results concerning those electrodes of interest for the hypothesized models and test thereof. For results concerning the entire EEG montage across the scalp, we refer the readers to the aforementioned published reports.

**Figure 5:**
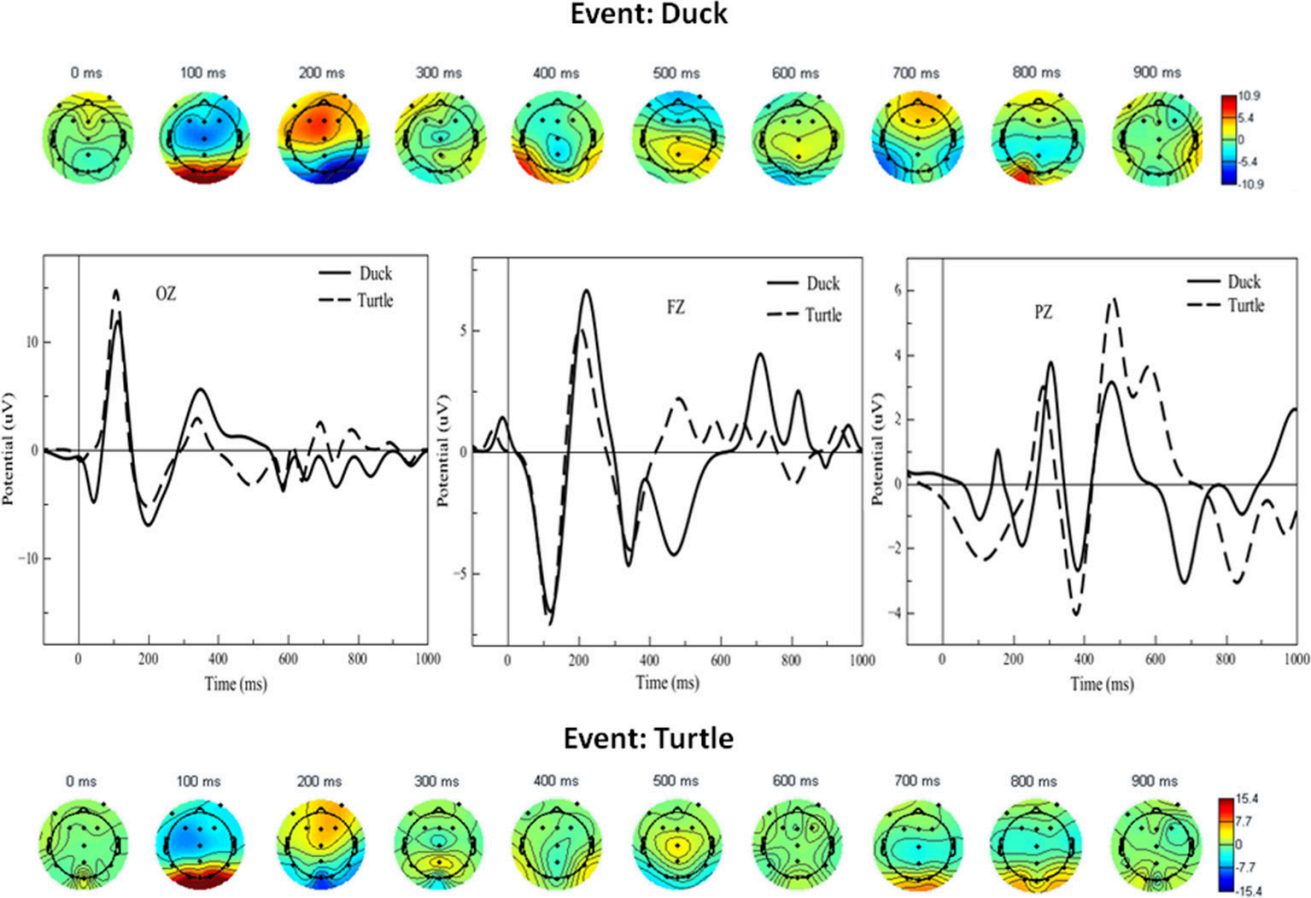
Bottom and top arrays: Scalp maps for selective attention task events (turtle, depicted with dashed lines, and duck, depicted with solid black lines). The graphs in the middle show ERP waveforms corresponding to the scalps at selected midline electrodes. Dark blue and red scalp areas represent significance at *p* < 0.05 in the Simes-Bonferroni corrected *T*-test band across the entire epoch (see text). We next focused our analysis on the two key proposed predictions. Figure 6B shows waveforms concurrent with the word-verification event for concrete and abstract sets of PPVT words, wherein the more concrete words included only items from the first set, and the more abstract included only items from the very last set successfully completed by each subject; given that the number of trials was reduced, the ERPs were smoothened to allow for a clearer evaluation of effects critical to this context. The most important and significant differences are highlighted in Figure 6C with gray frame boxes, for the site where the largest activation was detected through scalp maps, at the right occipital electrode (O2). As predicted, there was higher positive activation concurrently with concrete than with abstract words in the first 100 ms of the PPVT task [*t*_(13)_ = 6.52; *p* < 0.0001]. Conversely, there was higher positive activation concurrently with abstract than with concrete words in the 750–850 ms interval [*t*_(13)_ = 7.71; *p* < 0.0001].

For the attention task, grand averages of the ERP were calculated for the duck and turtle events, and the latter were then plotted as scalp maps. The array of scalp diagrams in Figure 5 shows the response for the duck (top) and turtle (bottom). The maps are views of the scalp from the top of the head and oriented with the anterior (frontal) areas at the top of the circle and the posterior (occipito-parietal areas) at the bottom. They are plotted as a function of time at every 100 ms. These provide a global dynamic view of the neural activity for all subjects during the tasks and therefore show several interesting features.

In the attention task, at 100 ms, both duck and turtle ERPs showed a high bilateral response in the posterior area, suggesting visual processing of the duck/turtle image. That was followed at 200 ms by strong frontal activity, perhaps indicating working memory determining the course of action. At 700 ms there was more bilateral frontal activity coinciding with the typical button press time. Focused contrasts revealed the strongest bilateral effects in the midline electrodes. The graphs in the middle of Figure 5 show the largest significant differences (the shaded areas in the figure) between attended (duck) and unattended (turtle) waveforms in the midline electrodes: between 300 and 500 ms in the occipital electrode, between 400 and 600 ms, and between 600 and 800 in the parietal electrode, and between 500 and 1,000 ms in the frontal electrode [all contrasts: *t*_(13)_ > 2.27, *p* < 0.05]. On average, the largest peak amplitude detected in the examined time windows was 28.4 μV (relative to baseline activity estimated at *Z* = 2.60, *p* < 0.01).

**Figure 6:**
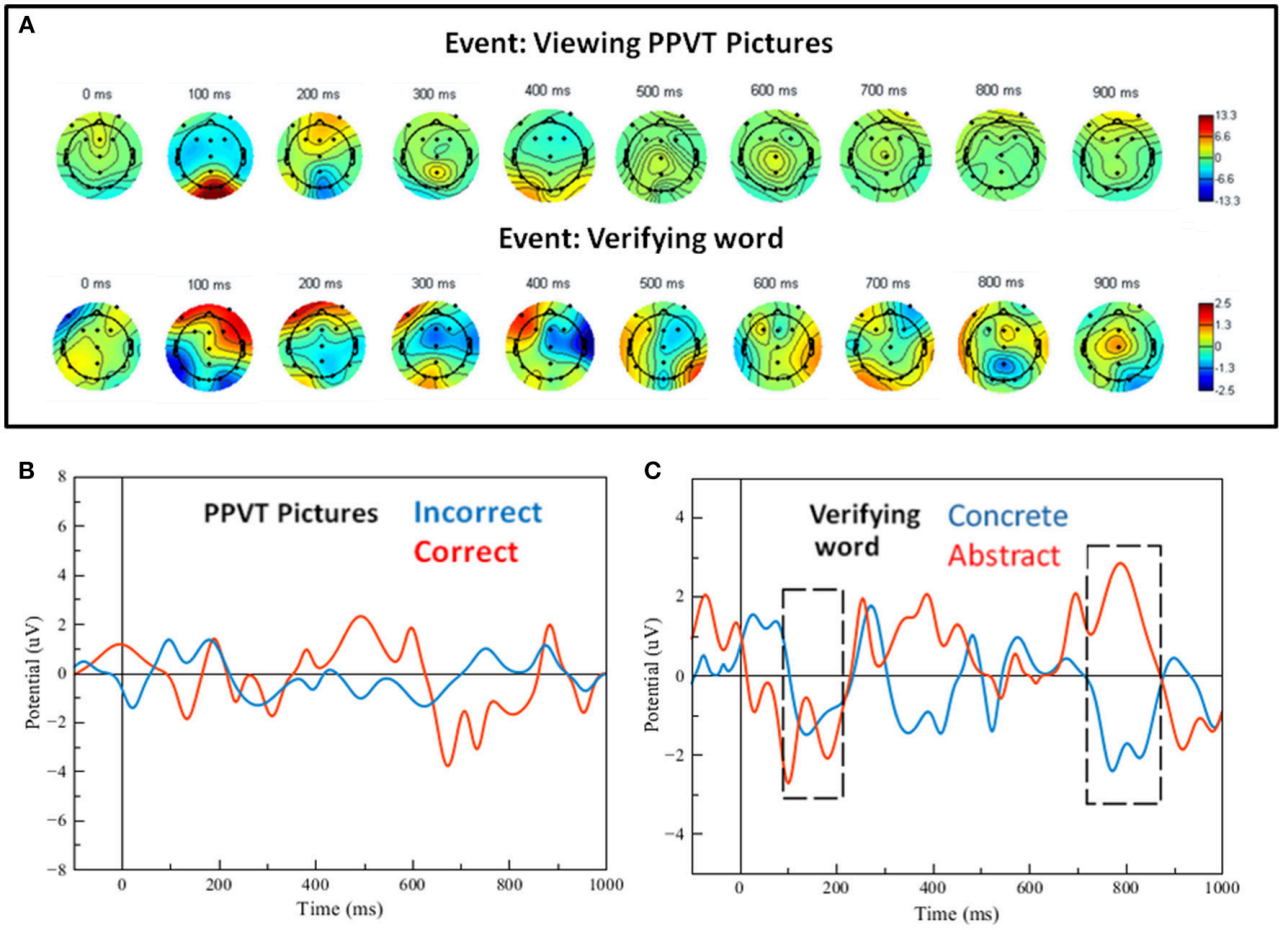
**(A)** Scalp maps for the word-verification task events (viewing pictures and verifying the word). **(B)** Filtered single-trial ERP waveforms of correct vs. incorrect word-verification trials at electrode FZ. **(C)** Filtered single-trial ERP waveforms for the verification of concrete and abstract words; the gray dashed-line frames show regions of significant effects (see details in text).

Grand averages for the ERPs of the PPVT were calculated and plotted as scalp maps as well. Figure 6A shows scalp maps for the event interval between presentation of the word and presentation of pictures (i.e., picture verification), and for the event of seeing the pictures display (i.e., PPVT pictures). Strikingly, the maps for ERPs concurrent to seeing the PPVT pictures are virtually identical to those observed concurrent to the viewing of the turtle—that is, the distracter event during attention (compare the bottom arrays of scalp maps in Figures 5, 6A against each other). Nevertheless, the maps for the word verification event show early co-activation of opposite polarity in right anterior and bilateral posterior electrodes from 100 to 200 ms. After 300 ms, the activity spread out mostly in the right side across the centro-parietal and temporal electrodes. To verify that deep elaboration occurred, the bottom panel of Figure 6 also displays (unfiltered) ERPs after hearing the target word, split by correct and incorrect PPVT trials, at Fz which was the most representative electrode. In the window between 400 and 900 ms, although the direction of the effect is reversed at about half of this interval, there is a significant difference between the waveforms of correctly and incorrectly identified words [the maximal effect is similar across, that is, a mean difference of about 5 μV, *t*_(13)_ = 4.23, *p* < 0.01].

**Figure 7:**
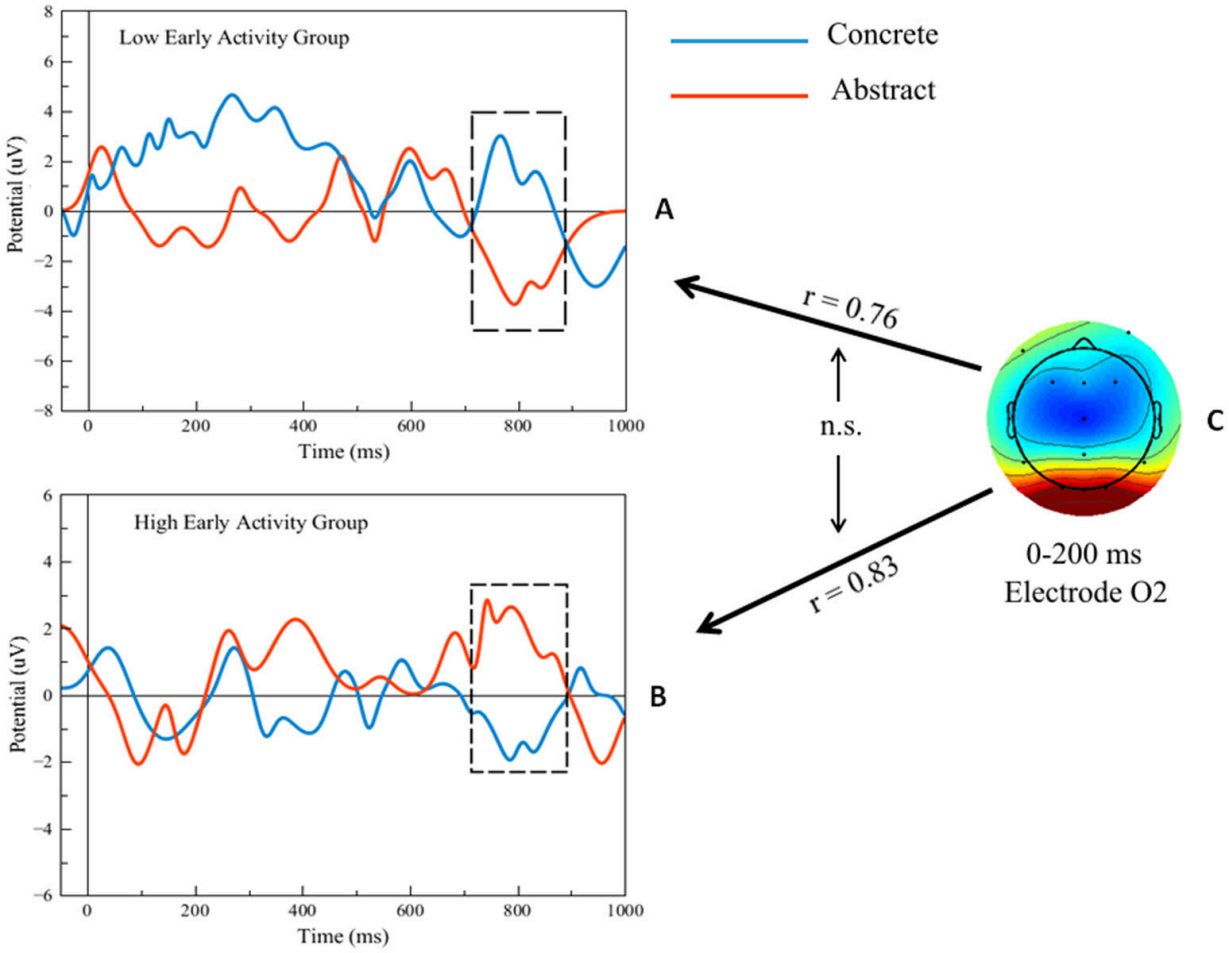
**(A,B)** Filtered single-trial ERP waveforms during the concrete vs. abstract word-verification task in high and low early activity groups. **(C)** Scalp map of single-trial ERP activity during the attention task and representation of predictions (black arrows) of the ERP differences in the regions shown by the black outline frames. Bidirectional black arrows represent statistical comparison (“n.s.”: non-significant).

The maximum value of the ERP voltage for each of the participants was computed between 0 and 200 ms at electrode O2 during the attention task, collapsed across correct duck and turtle trials. The participants were then subdivided into two groups based on the median split of the ERP voltage: one Low and one High *early activity group*; the PPVT concrete vs. abstract analysis was re-run separately for the latter two groups. The two graphs in Figure 7 show the ERPs at electrode O2, corresponding to PPVT event word-verification. The top graph shows the PPVT O2 ERPs for the seven subjects in the Low early activity group. The second graph shows the ERPs for the seven subjects in the High early activity group. In both graphs, the blue stands for the concrete PPVT word set (first set) and the red for the abstract (last set), as in the analyses presented previously. Confirming our second main prediction, at electrode O2 the late ERP activity (750–850 ms interval) was significantly higher for concrete than for abstract word verification in both groups [High: *t*_(13)_ = 4.00, *p* < 0.001; Low: *t*_(13)_ = 3.11, *p* < 0.01]. The effect size of the difference in late ERP activity between concrete and abstract word-verification was again predicted by the early ERP activity (0–200 ms interval) during the attention task. The individual variation of early perceptual/attentional processing predicted the variation of late activity related to word-verification, within the High early activity group (*r* = 0.83) as well as within the Low early activity group (*r* = 0.76). Importantly, when the effect sizes of the differentials of activity corresponding to concrete vs. abstract words were compared in the two groups (graphically represented by the bidirectional arrows in the right panel of Figure 7), this test did not yield significant differences (*Z* = 0.27; *p* = 0.79), showing that the level in early activity was similarly predictive of late activity in both groups.

### Control Comparisons Between Behavioral Data, Subject Data and ERP Data

Several multiple regression analyses were run to test possible associative relationships of ERP and EEG frequency bands (event-related band power analysis) with a host of control variables such as age, accuracy, and RTs on the tasks, as well as subjects' profiles used for screening and sample selection (CBCL, BRIEF-P, EDI). All effects were far from being significant (all p's > 0.50). This result showed that in the main findings we have reported above these other factors were not likely to be confounding.

### Modeling Data

Application of the ICA routine yielded from the experimental data between 8 and 13 separate components for ERP activity related to both attention and word-verification tasks, averaged across subjects. Only for a short period of time were the independent components resolved to be active. Their modeling was thereby facilitated as for separate minimally overlapping processes. The presence of each component was verified either by one or two dipoles through running the DIPFIT routine.

Simulation of EEG activity was achieved by construction of a computer model comprising eight modules, corresponding to those components found to be most prominent. When activated, each individual module was assumed to produce one or two dipoles lasting for its duration. Activation was modeled as a simplified spike, rising linearly to a peak and then dropping at the same rate. Dipoles were assumed to have been generated at the DIPFIT estimated location consistently with the locations assumed to be standard in ACT-R. The Talairach database was applied to map the corresponding regions of the brain (Lancaster and Fox, 2010). The models matched the distribution of experimentally measured potentials reasonably well. Overall, the models accounted for more than 75% of the spatial and temporal variation of electrical potentials; the model for dipole data fit expressed as R^2^ had a range of between 0.75 and 0.98—a fit of excellent quality when it is considered that the calculations contained many approximations and simplifications. An analysis of sensitivity suggested that measuring the scalp EEG voltage to ±10% precision would result in localizing a dipole within 2–3 mm. In particular, we were able to isolate three key processes associated with particular events during the tasks, which are detailed next.

**Figure 8:**
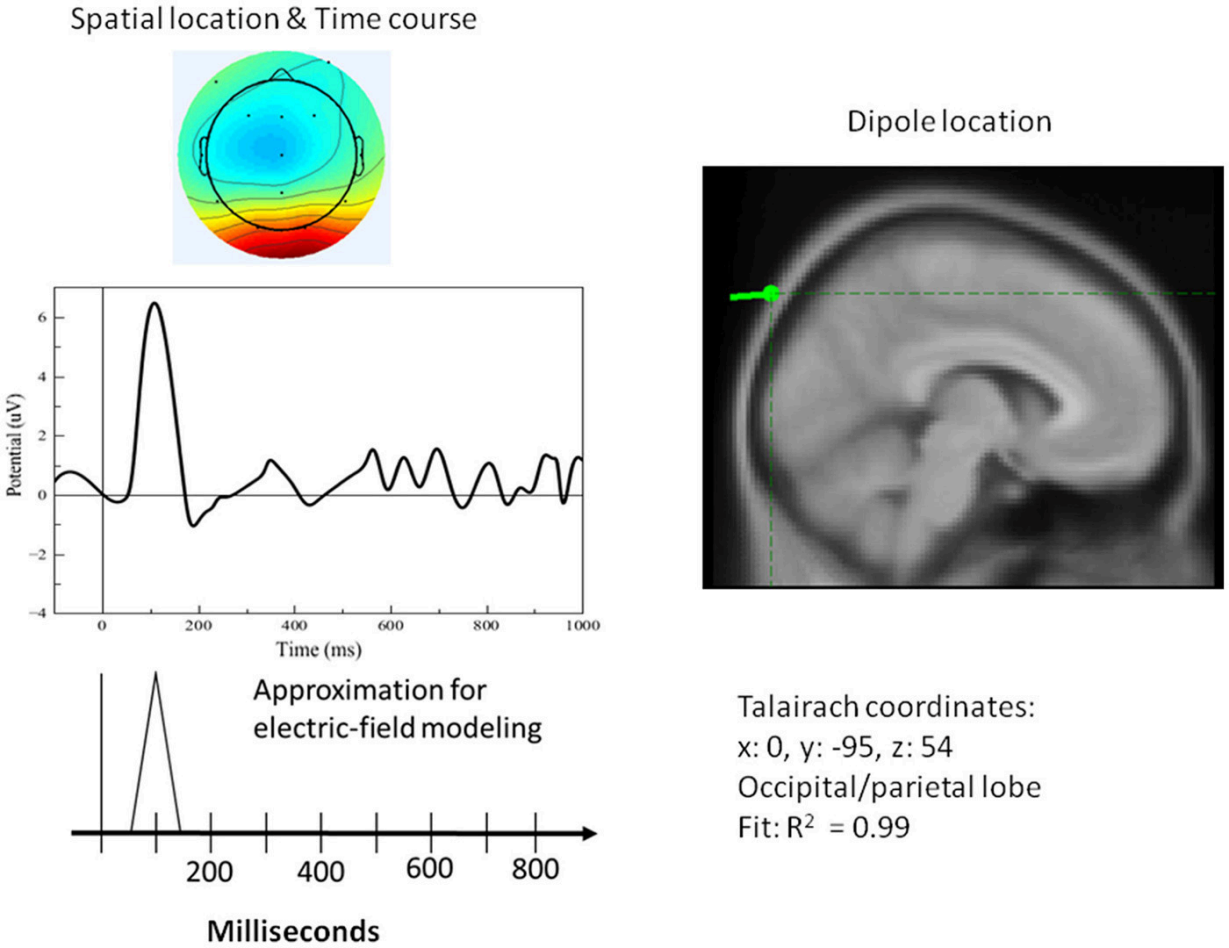
**Left top**: Spatial resolution and time course of the single-trial ERP component derived with ICA for early positive activity during the attention task for the target (duck). **Right top**: Dipole location. **Bottom Left**: Electric-field model of the component.

**Figure 9:**
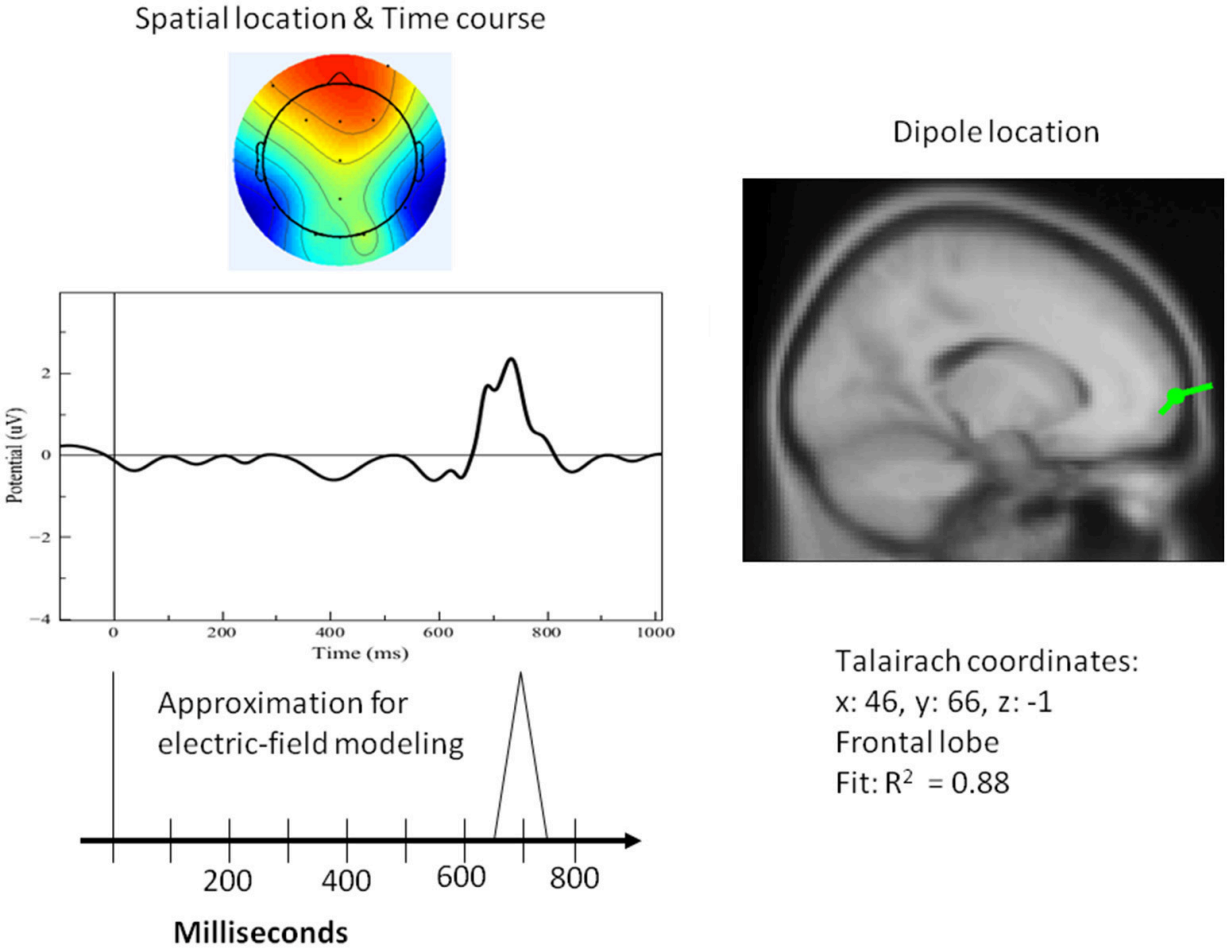
**Left top**: Spatial resolution and time course of the single-trial ERP component derived with ICA for late positive activity during the attention task for the target (duck). **Right top**: Dipole location. **Bottom Left**: Electric-field model of the component.

**Figure 10:**
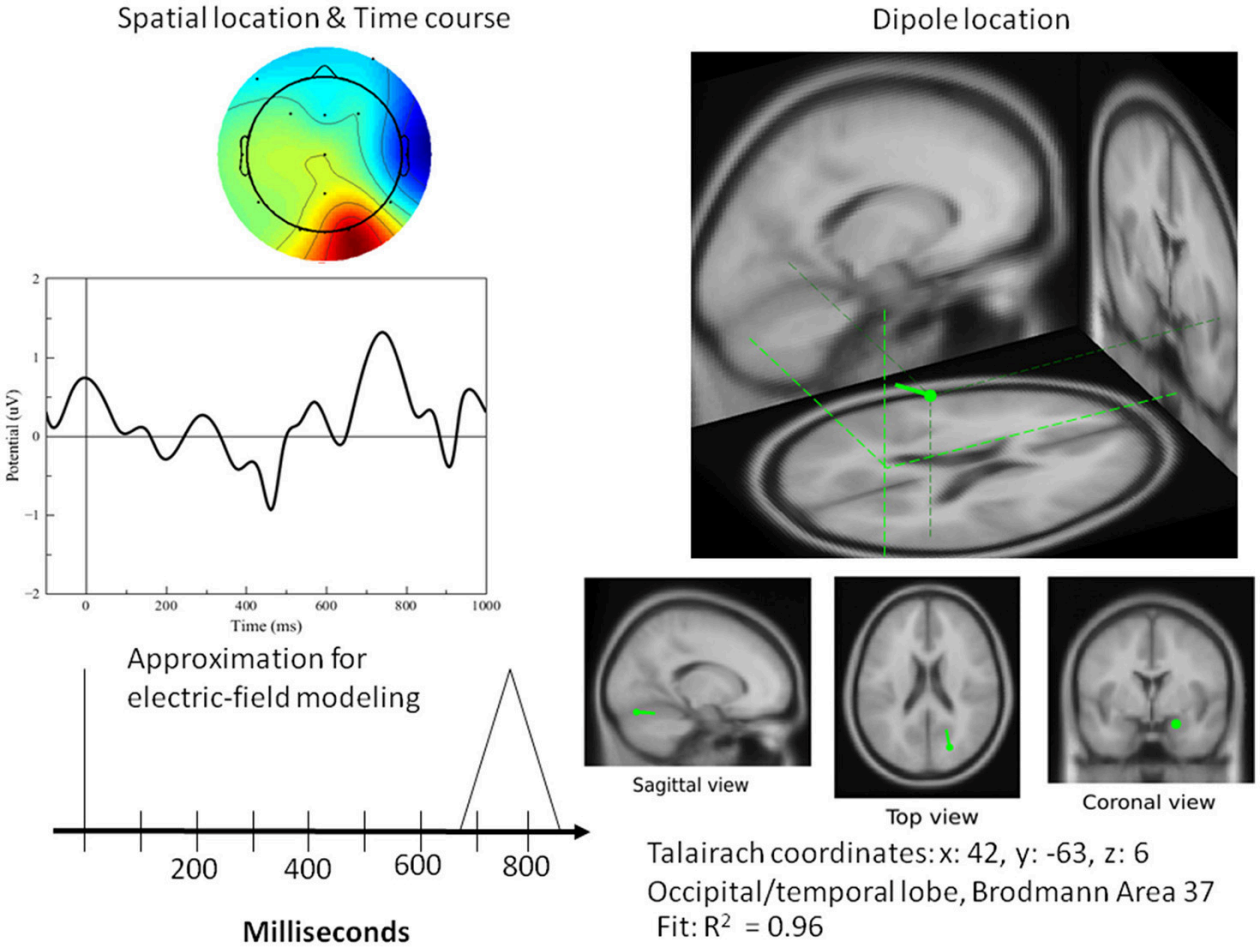
**Left top**: Spatial resolution and time course of the single-trial ERP component derived with ICA for late positive activity during the word-verification task. **Right top**: Dipole location. **Bottom Left**: Electric field model of the component. As far as the simulation results for the PPVT task (auditory-word perception followed by word verification process), Table 1 contains all the processes that were required to simulate the complete ERP signal for one epoch. Each line corresponds to one module within the cognitive model having the source location of one or of two electric dipoles. The times as they appear represent peaks in activity for every module.

Figures 8–10 present the most relevant results of the source analysis and simulation against the experimental results, specifically, during (1) perceiving and attending to the picture of the duck or turtle as well as the PPVT pictures, and (2) verifying abstract words. The top left-hand plots show contour lines of an ICA-derived single-trial ERP averaged over the subjects for the same target (the picture of the duck), but measured at different locations and at different times during the attention task. The views are from above, with the nose at the top of the diagram and the ears at the side. Darker areas indicate more positive voltage responses in the ERP. The bottom left-hand plots show the electric fields calculated from the model for those four modules. The times selected are the peaks of activity for those assumed to underlie the neurocognitive modules. On the right hand, the locations of the dipoles responsible for the fields are shown as small inverted pin symbols with lines indicating the orientation-positive polarity of voltage.

A single dipole explained the strong response at 100 ms concurrent with processing pictures in both tasks. The location of this dipole was in the posterior head in correspondence with the occipital areas as anticipated for attention and visual processing. The independent component in its time course produced a single spike at 100 ms, with negligible activity before it or after. It was therefore possible to model it in the form of a simple spike at 100 ms, reaching 50 ms on either side, and otherwise at zero (see Figure 8). Similarly, for verification of abstract words spike activity was isolated but deeper (see 3D representation in Figure 10) in the temporo-occipital area. For the attention task, another process was isolated in the frontal lobes with another clearly identified spike at about 700 ms (see Figure 9), the latter appearing appears quite distinct from late occipital responses.

**Figure 11:**
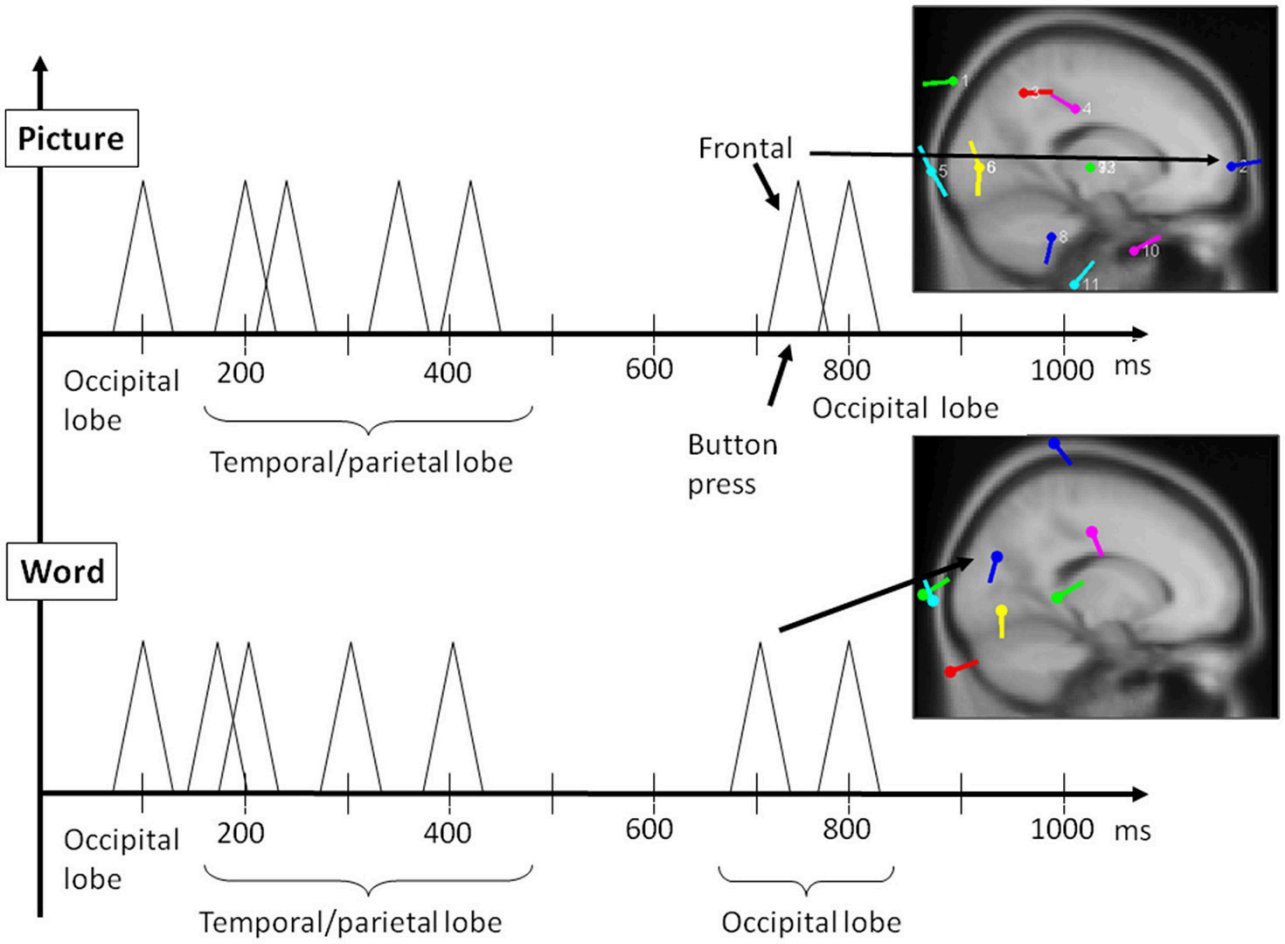
Reconstruction of most relevant ERP peaks as identified by ICA through electric field modeling for the two main phases of the PPVT task, auditory processing of the word **(bottom panel)**, followed by picture processing **(top panel)** (also refer to Table 1 above).

The output of the simulation closely reproduced the scalp electrical activity as measured in the experiment. In fact, the bottom panel of Figure 11 shows the electric field simulations next to the dipole analysis for the two types of events. The main three components we have described above are integrated with others in the simulation of the entire waveform. Importantly, two of these components, the early and late positivity, are observed during perception and word comprehension. The component representing the manual response shows clearly a different timing than that for late positive activity; this result is important because it rules out possible confounding factors between imagery and motor processes.

## Discussion

The present findings suggest that the EEG data of children can be simulated with use of a neurocognitive model, which assumes for each process that one to two electric dipoles are generated where the center of functionality is to be found for that function. Functionality mappings of ACT-R were proven to be robust in their use for EEG modeling. The standard locations of functions and the timings for productions were applicable. The experimental work described throughout this paper utilized results obtained from children which were first employed for the measurement of executive functions. Since the tasks may be considered relatively easy for children, the data may be considered optimal for the modeling of the cognitive processes of young children. Other future studies in modeling might make use of longitudinal results from the data of children and adults to offer a means of comparison in order to provide a test bench comparison of how modules may more specifically change throughout the course of development.

Adult neurocognitive modeling has usually been conducted for the purpose of the reproduction of averaged outward experimental behaviors of participants including response times and error rates. If, however, the objective is to simulate internal processes, it is advised that participants' differences (individual differences) be effectively considered. Our data of individual children showed that there were large individual differences in the processes taking place in the brain. For example, the activity in their pre-frontal areas revealed large variability between individuals (see Griffiths et al., 2009; D'Angiulli et al., 2010). These kinds of differences would have to be taken into consideration during any modeling specifically of individuals for personalized neurotechnologies (see the discussion as below). For example, ACT-R models usually only contain productions that are related to the task at hand. To reproduce the overall brain activity during the task, other processes such as environmental checks taking place in the brain need to be incorporated. It will therefore also be necessary to render tasks simple to ensure that consistent components be isolated for the sake of efficient modeling. Notwithstanding the general variability of EEG data, the technique of dipole analysis appears to be a very promising way to determine the localization, time course, and especially sequencing of neural events for the purpose of building increasingly complex neurocognitive simulations.

There are already links between ACT-R modeling and the learning sciences and education. For example, modeling based on fMRI imaging data has already been used to monitor children's mathematical learning procedures (e.g., Qin et al., 2004) and strategies (Tenison et al., 2014). However, the focus of these types of studies has mainly been to understand the processes underlying mathematical and arithmetic problem-solving during structured lab testing. In addition, research on ACT-R models has been used to build “cognitive tutors,” that is, computer-tutoring programs that implement the ACT-R architecture for the teaching of algebra, geometry, and integrated math (Anderson et al., 1995). The type of neurocognitive models described in the present study may be a further development in the possible application for learning and education by using ACT-R or other architectures for closed-loop brain training or, in other words, *educational neurofeedback* for practical uses with non-clinical populations and, in particular, with young children with or without special needs. In contrast to fMRI applications, EEG-based applications are relatively inexpensive, portable and wireless, and are more child-friendly in that they can tolerate to a certain extent some mobility and can be used in open spaces rather than a scanner. As a way of concluding the paper, possible future applications and research directions are discussed next with considerations for both progress and limitations of this research.

As discussed by Fairclough (2009), neurocognitive modeling could be integrated in closed-loop brain training to (i) induce the desired optimal psychological state prior to learning experience or examination (as suggested by sports neurofeedback, Hammond, 2007), to (ii) teach children about biological systems using biofeedback games (self-regulation exercises plus knowledge of human biology, see Sitaram et al., 2017), and to (iii) design adaptive educational software which in real time can keep the learner motivated, to avoid disengagement or boredom, as suggested by video games research (Lécuyer et al., 2008; Mishra et al., 2016; Kasemsap, 2017). Compatible with the traditional closed-loop brain training paradigm, in the first two instances (applications i and ii) the user works with the processed biological signal to develop a degree of self-regulation which can become a stable individual cognitive trait with practice (Sitaram et al., 2017). As far as it concerns the third instance (application iii), the software may be developed to be adaptive so that it may personalize and optimize the learning process for an individual. In other words, an efficient, optimal mind/brain state is being created on the fly by dynamic software adaptation to facilitate learning. It is unclear whether the latter application is as effective for encouraging self-regulatory strategies as traditional biofeedback, or whether it can serve as a potent tool for optimizing the learning process itself. The fact, however, that the neurocognitive modeling feedback is represented through content (simplified spikes) and that is congruent with the underlying brain processes might suggest that it could guide children to use natural mental strategies such as mental imagery (Scharnowski et al., 2015), which tend to be more successful in leading to learning and transfer.

Thus, in principle, it seems reasonable to speculate that EEG-based neurocognitive modeling of children's data could be used for indicating when/how teaching methods need to be revised, or as an assessment tool technique, monitoring how long children are engaged, interested or focused, as well as their actual understanding of materials and performance during the tests. EEG-based neurocognitive modeling may also help the teacher in assessing individual differences in specific aptitudes and preferences, demonstrating which learner has an aptitude for a subject or activity by the intensity, and patterns of activation in certain areas. It could therefore be used to assign students or groups of students to subjects or help them develop matching preferences to topics or projects.

Given that EEG patterns of young children differ from adults, a motivation for the current research was to model specifically children's data and use an approach that could lead to valid implementations. Accordingly, one of the implications is that the practical issues of recognition and classification of EEG patterns in young children can be minimized with increasing progress in the use of the approach. Because the technique demonstrated here can be designed so that models are not dependent on verbal or motor response, it could be used with young children—in particular, preschoolers. A future direction for research would be to adapt to extend the present approach to even younger children so that age is for any practical purpose virtually irrelevant. Single-trial ERPs could be measured routinely during learning, class or homework activity, and provided that one day the markers will allow early detection for learning difficulties, and especially reading and math disabilities. Suitable preventive teaching methods or early interventions could be put in place before the problems start having their negative effects. Importantly, it may be possible to detect or confirm clinical conditions such as ADHD by examining EEG activity showing, for example, the relative ratio of beta and theta EEG frequencies generated in certain ROIs in the brain.

In addition to the potential usefulness for diagnostic purposes, a better specification of the neural dynamics through EEG-based neurocognitive modeling may afford to describe the learning process more accurately, and to adapt teaching accordingly. In video games research, some applications are already enabling computer activities to respond to the affective states of the user classified as being, for example, bored, angry, excited, or confused. This approach can be directly transferred into education environments where information, activities, tasks and exercises can be tailored at an appropriate level and in an enticing way. Furthermore, the feedback the system uses about the person's state could become part of neurofeedback itself, as it could be used for supporting practice and training of key aspects of executive functions and attention.

It seems plausible that personalized neurofeedback in education likely will not be a stand-alone, but one of many tools for the learners. An interesting approach would be to integrate EEG-based neurocognitive modeling in systems that use multiple techniques over time for changing behavior (see NASA PlayAttention; Palsson and Pope, 2002). This application could be similar to those used to enter the optimal mind/brain state or to improve performance by practicing to generate relevant EEG timing, patterns and intensities at certain frequencies, or through learning interfaces in which desired states are elicited indirectly.

The present research though is only an initial step toward the development of the learning environments hypothesized above. At this point, EEG-based neurocognitive modeling only supports a relatively reliable identification of generic states; personalization will require many layers of improvement and fine tuning to allow monitoring to identify an individual state and then training to replicate it consistently and reliably. The present research opens up many interesting questions for future research regarding knowledge representation in educational neurofeedback. Can neurofeedback based on the simplified spike representation as shown here with EEG-based neurocognitive modeling be a suitable method of communicating information? And can it be used as an appropriate way for ensuring that information is presented meaningfully to young children? It is unclear at this point if it is more sensible to use such an approach to engage and monitor rather than to teach *per se*. Many questions hinge on the type of interface for education. Should it relate directly to a subject such as human biology, as discussed earlier, or can it be more abstract, and simply inducing the desired mind/brain state could be sufficient? Can shared cooperative or competitive environments be created to be more engaging to achieve the desired state? Also, many other very important related practical aspects such as aesthetics (the appearance of the interface and electrodes), wearability, mobility/portability, and degree of invasiveness are in need of much more future research.

All analyses, simulations and modeling included in this paper involved a novel combination of existing, commercially available or even open source tools that are widely available and used in the research and consumer communities. Therefore, a third implicit goal of this paper was to show that the approach used here, or a similar approach, could in principle be adopted relatively easily by non-experts in educational and pedagogical settings. However, it is important to point out that there are limitations in terms of *reliability*, that is, precision of measurement afforded by the existing tools we used. The particular implementation of ACT-R used in this study is based on a spherical head assumption, which makes possible mapping ERP activity onto MRI-based anatomo-functional structures. However, the extraction of ERP data under the sphericity assumption employs the common average across the scalp as the reference channel for the recorded EEG data. Other reference methods, specifically the reference electrode standardization technique (REST) (Yao, 2001), have been shown to give more precise temporal information on EEG recordings. However, even with the current limitations of the tools, the approach we used seems reasonably robust in terms of the validity of neurocognitive modeling, because it focused on insuring that those tools identified and measured the internal processes as predicted. Ultimately, the validity was insured by comparison with a body of ACT-R modeling work, which has been done in both adults and children. We are not aware of currently widely available EEG-based ACT-R applications in children that used REST. Therefore, we suggest that an important extension of the present work is for future research to devise ways to assess the validity and reliability of REST in ACT-R neurocognitive modeling of children's data, thereby establishing a base of knowledge and use for the benefit of non-experts.

In conclusion, the retooling of existing computational techniques in novel ways such as the one demonstrated by our study opens a host of possible innovations in neurotechnological applications for personalized and inclusive education. Although we are still far from actual effective implementation of credible and dependable neurotech for learning, thinking about their potential and how we will use them and when they will be ready is crucial so that education tools can be properly designed from inception. All stakeholders (teachers, students, academics, and parents) need to be involved at the onset—that is, from now—as part of research development to create systems that are useful, usable and meet the highest ethical and safety standards.

## Author Contributions

AD was responsible for all aspects of manuscript. PD was responsible for critical revision and editing of all manuscript text after the first independent review, and re-analysis of data; redrawing and enhancing all figures, and helping with final editorial tune-ups, and checking of references.

### Conflict of Interest Statement

The authors declare that the research was conducted in the absence of any commercial or financial relationships that could be construed as a potential conflict of interest.
